# The Benefits and Risks of the Provision of a Hospital-Wide High-Definition Video Conferencing Virtual Visiting Service for Patients and Their Relatives

**DOI:** 10.7759/cureus.13435

**Published:** 2021-02-19

**Authors:** Adeel Abbas Dhahri, Agampodi Umanda De Thabrew, Nirali Ladva, Helen Pardoe

**Affiliations:** 1 General Surgery, Princess Alexandra Hospital NHS Trust, Harlow, GBR; 2 Emergency Medicine, Princess Alexandra Hospital NHS Trust, Harlow, GBR; 3 Physician Associate, Princess Alexandra Hospital NHS Trust, Harlow, GBR

**Keywords:** quality improvement projects, virtual visits, video telemedicine, covid-19

## Abstract

Background

Since the start of the coronavirus disease 2019 (COVID-19) pandemic, virtual visiting (VV) has become important because of visiting restrictions in hospitals. This project aimed to determine the impact of VV on staff and patients’ loved ones (visitors).

Methodology

VV is defined as high-resolution video communication between admitted COVID-19 patients and their loved ones in the presence of a staff member using a healthcare platform. VV was introduced in a 419-bedded hospital in the UK in April 2020. Qualitative data on the VV experience were collected from relatives and staff via an open feedback email address and reflective practice. Data were entered and analyzed in person by two independent assessors. Grounded theory methodology and thematic analysis were used to draw conclusions.

Results

Between April 16, 2020 and November 30, 2020, 1,009 visits were delivered. There were 138 feedback responses; 108 (78.3%) from relatives and 30 (21.7%) from staff. The amalgamation of data was resolved into five themes: appreciative factors (129, 93.5%), organizational skills (44, 31.9%), palliative care (38, 27.5%), staff communication (14, 10.1%), and VV process issues (11, 7.9%).

A total of 131 (94.9%) responses had positive comments (111 from relatives, 20 from staff); negative comments were greater in the staff cohort (23%) than the relative group (4%). Trends included sub-themes in overwhelming emotions, emotional strain for staff members, and difficult situations.

Conclusions

VV in hospitals is a new and valuable way to connect patients with loved ones with mostly positive consequences. VV also has risks to mental health and well-being, particularly for healthcare workers facilitating the call.

## Introduction

Since the onset of coronavirus disease 2019 (COVID-19), the world has witnessed a rising infection, rate and in September 2020 a new variant of the virus was identified in the UK. This variant is considerably more infectious than the original variant and is spreading rapidly across the world [1]. The World Health Organization (WHO) continues to urge social distancing as one of the primary preventive measures for this pandemic [2]. For any country aiming to control the spread of the new variant in the healthcare environment, consideration will need to be given to discontinuing all hospital visiting, even in the end of life (EoL) scenario. Additionally, international travel is again being significantly curbed, which further distances patients from their relatives and loved ones. The COVID-19 pandemic has left significant psychological effects on the population with increased levels of loneliness and depression, not only in the general population but especially among healthcare workers [3].

Since WHO declared COVID-19 as a pandemic, the field of medicine has rapidly evolved and transformed, including the widespread use of telemedicine. With the universal introduction of social distancing, for the safety of both patients and healthcare workers, telemedicine has been successfully incorporated into the healthcare setting. Telemedicine has a spectrum of provision, ranging from text messages and telephonic interaction to video consultation as a replacement for face-to-face consultation. Such provisions have been utilized not only in the outpatient areas but also within the hospital wards as virtual visiting (VV) [4-6]. VV in this paper is defined as high-resolution video communication between admitted COVID-19 patients and their loved ones in the presence of a staff member using a dedicated healthcare platform. Further emphasis has been put on VV since the start of the COVID-19 pandemic when family and friends’ in-person visits to hospital patients were canceled across the world [6,7]. Devices like tablets or iPad can be used while being fixed to a pole, for example, near the isolated patient for communication as a video conferencing tool [8]. However, many patients are too elderly or too unwell to interact independently with a device and there is a need for a healthcare worker to facilitate communication. Another factor to consider is the cybersecurity associated with a hospital domain, meaning that popular commercial solutions such as FaceTime, House Party, or Zoom may not be available on the hospital devices [9].

With the rise in psychological distress around the world, related to the COVID-19 pandemic, VV has come to the forefront with positive and promising outcomes across the globe. The general population and healthcare workers have widely accepted VV in patient-centered care [3,6,10]. However, there is little published literature on the possible negative effects of delivering a VV service in the hospital setting. Given this background, we aimed to determine the impact of VV on staff and family members or loved ones, the virtual visitors, of patients affected by the COVID-19 pandemic with the resultant transferability of this technology in the future.

## Materials and methods

The VV service was introduced into the hospital using a rapid quality improvement approach using the Institute of Healthcare Improvement methodology initiated on March 31, 2020 with a successful go-live date of April 16, 2020 [4]. The VV was delivered as a video call between an admitted COVID-19 patient and their family member or loved one in the presence of a member of staff, using a cyber-secure user-friendly software, Attend Anywhere (AA), on a digital equipment (iPad). This service was organized in five dedicated COVID-19 wards through an email platform for relatives of admitted patients. Family members who wished to see their relative on the same day could request a VV through email run by an administrative staff team.

A standard pperating procedure (SOP) was described and briefed to the staff members. Dedicated and passionate volunteers were recruited from a pool of existing hospital staff and physician associate students. Using hospital iPads, volunteers were asked to carry out a 10-minute video call with the patient in full personal protective equipment (PPE). The team of staff received a script detailing their role and how to carry out calls as per Appendix 1. Each patient had to be identified by ward staff and confirmed that VV was appropriate. Volunteers were required to seek out the visitor’s knowledge of the patient’s condition before the patient joined the VV. Giving personal medical advice or information was prohibited. Following the call, volunteers entered the VV details in the integrated healthcare records. There was no formal survey questionnaire. The visitors and staff were encouraged to provide email feedback in the form of reflection of their experience after each call. All these email responses were confidential and received via the VV email platform.

The unaltered transcripts of the email responses from staff and family members were checked to ensure variations in responses to avoid duplication. They were then coded manually according to personality type, positive or negative response, the clinical status of the patient, and time period using the grounded theory methodology [11]. A single doctor coded the data and independent data validation was performed by a second analyst. Thematic analysis was used to draw conclusions from the data.

The introduction of the VV service passed through local governance processes which had been streamlined in response to the urgent needs of the emergency situation. This study has been reported in line with the Standards for Quality Improvement Reporting Excellence (SQUIRE 2.0) [12].

## Results

A total of 1,009 VV were delivered within the time frame described. There were 138 feedback responses, including 108 (78.3%) from relatives and 30 (21.7%) from staff. A total of 27 codes assigned to the data were amalgamated into the five stated themes. There were five final themes identified from the data following analysis: appreciative factors (129, 93.5%), organizational skills (44, 31.9%), palliative care (38, 27.5%), communication from staff (14, 10.1%), and VV issues (11, 7.9%). Overall, 94.9% of the total responses had generally positive comments (111 from relatives and 20 from staff):

“Thank you so much to you and all the team for making this valuable service available to us and doing a great job, all really appreciated it.”

“Dear virtual visiting team, much appreciated for all your efforts. It’s an amazing service and we’re very grateful, Thanks.”

Appreciative factors

There was a total of 129 comments identified under four sub-themes for appreciative factors: 41 (29.7%) were generally positive comments, 46 (33.3%) related to family closeness, five (3.6%) to life events, and 37 (26.8%) to staff care (Table 1).

**Table 1:** Feedback themes and their sub-themes. VV = virtual visits

	n	%
1. Appreciative factors		
Positive feedback	41	29.7
Family closeness	46	33.3
Life events	5	3.6
Staff care	37	26.8
2. Organizational skills	44	31.9
3. Palliative care		
Overwhelming emotions	11	7.9
Closure	4	2.9
Time with relatives	9	6.5
Support	7	5.1
Shocked with patient condition	7	5.1
4. Staff Communication		
Emotional strain for staff	5	3.6
Difficult situation	7	5.1
Communication with ward staff	2	1.5
5. VV issues		
Medicalization of the VV	0	0.0
Family did not attend	4	2.9
Using feedback portal as request for VV	7	5.1

Responses commented on being able to celebrate birthdays and anniversaries together through VV and the usefulness of VV allowing more than one family member to join the call:

“How lovely my darling to have seen you on the iPad yesterday on your birthday please thank the ward staff and all those other marvelous ladies for helping us to sing happy birthday, we were all thrilled that you were able to remember all our names.”

“It was amazing to see you yesterday and singing Happy Birthday to you.”

“My Grandmother and I loved to have an opportunity to have a video call with Grandfather.”

Organizational skills

Table 1 shows that 44 (31.9%) relatives mentioned that staffs’ organizational skills were excellent, which shows how well a VV service can run in a district general hospital.

Palliative care

For the patients who were near the EoL, there were four sub-themes identified, including overwhelming emotions (11, 7.9%), closure (4, 2.9%), time with a relative (9, 6.5%), and support (7, 5.1%) (Table 1). We also identified negative remarks concerning the palliative care theme. A total of seven (5.1%) comments confirmed that sometimes family members were shocked even if they had been warned that their relative was very unwell:

“The patient is too unwell to speak ... and it was too difficult for them both.”

However, there is clear evidence that VV allowed family members to maintain a connection with the patient before EoL. This seems to have been of particular significance to the “visitor.” Support by staff through follow-up calls or visits and communication from staff was particularly praised:

“XX was extremely grateful for the follow-up call (EoL family member).”

“The two virtual visits that took place were arranged and delivered through a very empathetic and accommodating team.”

Communication from staff

The three running sub-themes for staff communication were an emotional strain for staff members (5, 3.6%), difficult situations (7, 5.1%), and communication with ward staff (2, 1.5%). Staff remarked on the sadness of some VVs (9, 6.5%) (Table 1). Although volunteers were not required to give any medical advice, the nature of the role meant they sometimes were required to discuss and witness difficult topics. There was some miscommunication at times. One volunteer discussed:

“This afternoon XX had deteriorated, and the family was made aware by the ward staff … The call started and very quickly into the call they were very shocked and upset to see him so unwell. I then asked the family if they would like a break and they said that it was enough for today.”

Virtual visit issues

There was no indication of the medicalization of the visit by attendees. Unfortunately, some families did not attend (DNA) sessions (4, 3.9%) or tried to organize visits through the reflection feedback emails (7, 5.1%) (Table 1).

## Discussion

The COVID-19 pandemic effects have changed hospitals’ infection control measures, including social distancing and reduction of footfall [13]. For the hospitalized patient, this means no visitors are allowed. For relatives and friends (visitors), this results in a lack of physical and emotional contact, with social distancing leading to the risk of psychological harm. This psychological effect snowballs into a sense of desperation for the visitors wanting to be with their loved ones admitted to the hospital. The lack of physical visiting further adds to a sense of helplessness and guilt that has not previously been experienced in healthcare [14]. Although the healthcare teams keep visitors updated about patients’ clinical progress, it is not a substitute for a physical visit. The COVID-19 pandemic provided the opportunity based on the need for the rapid deployment of existing technology, and physical visits from visitors were replaced by VV. VV is now available in many hospitals in the UK [6]. The choice of the video conference platform needs to be made with consideration for healthcare cybersecurity and patient confidentiality, making some widely used social media platforms an unsuitable choice for VV.

Literature and media coverage has shown that VV has given families and loved ones a way to enjoy live events together when COVID-19 restrictions have restricted face-to-face visiting. VV also helps visitors connect with patients even during difficult times such as EoL. VV has enabled visitors to connect with their loved ones-each person can see the expressions on their faces, hear their laughter, hear their concern, and hear their love [8]. The overwhelming support for VV has been based on the appreciative factors identified by frontline clinical staff, especially those in the critical care setting [6,10]. This has encouraged the widespread adoption of VV as an addition to patient care. In our study, a small number of visitors were shocked at seeing the patient’s condition and did not want to continue the VV. Previous studies have shown that there is an emotional toll for families and relatives of VV, and the data presented here reflect similar issues [6]. Overall, our study found positive and encouraging feedback from patients’ relatives and friends in response to organizational and communication skills. The suitability of patients for VV should always be assessed by the staff with the help of the ward team before arranging such a service.

It is of note that our data show that the staff facilitating the call may be at greater risk than the visitors from long-term psychological harm from VV, particularly when patients are receiving ward-based EoL care. Staff facilitating the calls experienced distress seeing relatives who were upset. At times, the facilitators were observers to people saying a final farewell to a loved one, and this was identified as another situation that could affect the well-being of staff [15]. In our study, during such cases, visitors reported that VV had helped them feel close to their loved ones before EoL.

The hospital staff’s communication skills are an integral part of the VV service for the visitors who require to be properly briefed before starting a VV to overcome unnecessary psychological after-effects when they are already in a state of heightened emotion.

To mitigate the risk of psychological harm to staff that could result from the introduction of a VV service to a hospital, the hospital would be advised to develop an SOP, support the team with a recommended script for the VV, and have a systematic organizational approach to service delivery. We would also recommend regular debriefing sessions for staff to help build an effective and productive team, as well as a training program and mentorship to support the staff delivering the VV. During the first wave of the pandemic, the staff was supported with a weekly debrief session which moved to a monthly session as the hospital returned to business as usual. With such a coordinated approach, emotionally fragile visitors may find the VV a more sympathetic life event and experience less stress, and the risk of psychological harm to the healthcare staff is minimized.

Like any deployment during a crisis, VV is not without obstacles and issues. When virtual telemedicine (AA) was started in our hospitals, despite its dynamic applicability and adoptability, weaknesses like internet lag and audio and video quality problems, especially when the staff was wearing PPE, were also identified [4]. Where the patient cannot communicate or where the EoL process has been started, challenges can be faced. Furthermore, it was found in our study that relatives and friends sometimes DNA a scheduled virtual visit. This could cause distress to the patient, reduce opportunities for others, and potentially could delay requests for further VV.

VV has overall been a success at our hospital by providing patients and their loved ones with sympathetic support during difficult and isolating times. We recommend continued VV while working closely with the ward staff. There should also be detailed training and support for VV staff before and after visits to provide a smoother experience. Adverts for the VV service should emphasize issues with DNA and asking for any medical updates. With increasing technology and older populations, this scheme helps to marry the two and provide patients with familiar connections during the pandemic, which could also be applicable in routine practice. We recommend conducting further surveys with more data from patients and the current sample group in the future to support, improve, and develop VV for all stakeholders.

We do understand that despite a large number of visits, the responses were low. We assume that staff were busy with dedication in their duties during the COVID-19 pandemic crisis. This may reflect the limitation of our study.

## Conclusions

VV, the digital healthcare solution delivered to the front line has worked effectively with excellent reliability in a cyber-secure environment. The risk of the technology comes if hospitals introducing a VV service fail to have in place psychological support systems for patients, visitors, and staff to mitigate the risks of long-term psychological harm resulting from the introduction of technology and new ways of working. Despite these barriers or weaknesses, VV has the potential for widespread implementation in the hospital wards even in the post-pandemic era.

## Appendices

Virtual visiting script

1.     Ensure you have personal protective equipment (PPE) and red scrubs on before seeing any patient

2.     Speak to the ward manager and the medical team looking after the patient

3.     Introduce yourself, that you are with the patient experience team and therefor a virtual call

4.     Ensure the patient is fit and well enough to make the call and interact with the family

5.     Be sure to ask the medical team if the family is aware of the patients’ condition

6.     Speak to the patient and ensure they are happy to take the call from their family

7.     Put your PPE on

8.     Accept the call from the waiting room and let the family know the following;

a.     Introduce yourself

b.     Confirm who they are here to see

c.     They have roughly ten minutes to have a chat

d.     Let the family know that if they feel too distressed or want to stop at any point to let you know

e.     If the patient is unable to speak or very unwell, speak to the family and explain that their family member is quite poorly and may not be able to speak back/interact but will be able to hear them. Explain there is no pressure to see them if they do not feel up to it

f.      Ask them if they would still like to see them-if yes, take them through

9.     As time goes on, most of the time the conversation will come to a gradual end-facilitate goodbyes and wish the family all the best and end the call

10.  Remove PPE

11.  Wash hands

12.  ClinelÔ wipe the iPad

13.  Write in the patient notes that the virtual visit took place and any other relevant information

NB: You are not here to give any clinical information out:

                 If the family ask for a medical opinion, inform them that you are not part of the medical team looking after the patient and that they can call the ward up and they will be put through to the appropriate person.
